# Genetic Analysis of Cold Tolerance at the Germination and Booting Stages in Rice by Association Mapping

**DOI:** 10.1371/journal.pone.0120590

**Published:** 2015-03-19

**Authors:** Yinghua Pan, Hongliang Zhang, Dongling Zhang, Jinjie Li, Haiyan Xiong, Jianping Yu, Jilong Li, Muhammad Abdul Rehman Rashid, Gangling Li, Xiaoding Ma, Guilan Cao, Longzhi Han, Zichao Li

**Affiliations:** 1 Key Laboratory of Crop Heterosis and Utilization, Ministry of Education, Beijing Key Laboratory of Crop Genetic Improvement, China Agricultural University, Beijing, 100193, China; 2 Institute of Crop Science, Chinese Academy of Agricultural Sciences, the National Key Facility for Crop Gene Resources and Genetic Improvement, Key Laboratory of Crop Germplasm Resources and Biotechnology, Ministry of Agriculture, Beijing, 100081, China; 3 Rice Research Institute, Guangxi Academy of Agricultural Sciences, Nanning, Guangxi, 530005, China; International Rice Research Institute, PHILIPPINES

## Abstract

Low temperature affects the rice plants at all stages of growth. It can cause severe seedling injury and male sterility resulting in severe yield losses. Using a mini core collection of 174 Chinese rice accessions and 273 SSR markers we investigated cold tolerance at the germination and booting stages, as well as the underlying genetic bases, by association mapping. Two distinct populations, corresponding to subspecies *indica* and *japonica* showed evident differences in cold tolerance and its genetic basis. Both subspecies were sensitive to cold stress at both growth stages. However, *japonica* was more tolerant than *indica* at all stages as measured by seedling survival and seed setting. There was a low correlation in cold tolerance between the germination and booting stages. Fifty one quantitative trait loci (QTLs) for cold tolerance were dispersed across all 12 chromosomes; 22 detected at the germination stage and 33 at the booting stage. Eight QTLs were identified by at least two of four measures. About 46% of the QTLs represented new loci. The only QTL shared between *indica* and *japonica* for the same measure was *qLTSSvR6-2* for SSvR. This implied a complicated mechanism of old tolerance between the two subspecies. According to the relative genotypic effect (RGE) of each genotype for each QTL, we detected 18 positive genotypes and 21 negative genotypes in *indica*, and 19 positive genotypes and 24 negative genotypes in *japonica*. In general, the negative effects were much stronger than the positive effects in both subspecies. Markers for QTL with positive effects in one subspecies were shown to be effective for selection of cold tolerance in that subspecies, but not in the other subspecies. QTL with strong negative effects on cold tolerance should be avoided during MAS breeding so as to not cancel the effect of favorable QTL at other loci.

## Introduction

Low temperature is one of the major abiotic stresses that threaten the adaptability of rice and its production. Due to the long growing season and frequent low temperature in North China, Korea, Japan and other countries at high latitudes, the growth duration of cultivars that cannot tolerate low temperature has to be shortened and this usually results in low yields. Cold stresses that often occur during flowering and grain filling all over the world [1–2] may also lower grain quality and yield. Cold tolerance of rice is a complex quantitative trait significantly influenced by environment. Thus research on the underlying genetic mechanisms and discovery of genes affecting to cold tolerance in rice will be helpful for developing elite cultivars with strong cold tolerance.

Segregating populations are usually used for mapping and cloning genes for cold tolerance. More than 30 quantitative trait loci (QTLs) for cold tolerance have been mapped on 12 chromosomes (according to www.gramene.com). Most of them were for cold tolerance at the germination stage, and a few were cloned. Using backcross inbred lines of Livorno/Haymasari, the QTL *qLTG3–1* was cloned and proven to contribute to the high germinability under low temperatures [3]. Mapping QTL for cold tolerance at the booting stage is more difficult because of difficulties in phenotyping and the underlying complex biological and genetic mechanisms involved. Eight QTLs related to spikelet fertility under cold stress were mapped on chromosomes 1, 4, 5, 7, 10 and 11 based on NILs derived from KMXBG as donor and Towada as the recipient [4–5]. Saito et al. mapped the QTL *Ctb1* encoding an F-box protein and contributing to normal anther development under cold stress [6].

In contrast to traditional QTL mapping, “association mapping” is an alternative approach for studying complex traits. Association mapping, also known as linkage disequilibrium mapping, utilizes allelic variation in natural populations, and is capable of simultaneously identifying many loci for multiple traits [7]. Association mapping has been employed in rice to identify QTLs or genes related to yield [8–10], heading date [11], disease resistance [12–13] and tolerance to abiotic stresses [14].

Using 174 rice accessions from the mini core collection of cultivated rice and 273 simple-sequence repeat (SSR) markers we detected QTLs associated with cold tolerance during the germination and booting stages. We subsequently screened the accessions with strong cold tolerance and QTL markers showing strong effects, which will be the potential parents and markers in developing cultivars with cold tolerance by molecular marker assisted selection.

## Materials and Methods

### Plant materials

The research material comprised 174 diverse accessions from the mini core collection of cultivated rice [15]. Among them, 118 were landraces, 56 were modern improved cultivars and 109 were subspecies *indica*, and 65 were *japonica* (S1 Table).

### Evaluation of cold tolerance at the germination stage

Fifty seeds of each accession were placed in a drying oven at 50°C for 48 h to break dormancy, and then soaked in distilled water in a 15 cm Petri dish for 24 h. The pre-soaked seeds were germinated in a growth chamber at a constant temperature of 32°C for 36 h. Germinated seeds with 5 mm coleoptiles were stressed at 5°C for 10 days, and then moved to a greenhouse at 20°C for 10 days to allow seedlings to recover and resume normal growth. Seedling survival rates (SSvR) were then scored as a measure of cold tolerance at the germination stage.

### Evaluation of cold tolerance at the booting stage under naturally low temperature conditions

One measure of cold tolerance at the booting stage is the seed setting rate under naturally low temperature conditions (SStR-NL). Suitable conditions are available at an experimental farm of Yunnan Academy of Agricultural Sciences, Kunming, where atmospheric temperatures are 15–19°C during the booting stage extending from June to August [16]. Seedlings of each cultivar were grown in a nursery seedbed planted 31 March 2006, and transplanted on 9 May with two replications. Twenty plants of each accession were transplanted in a single row with a row × plant distance of 25 × 15 cm. The field was fertilized with N (120 kg/ha^2^) and P_2_O_5_ (80 kg/ha^2^). SStR-NL was scored as the mean seed setting rate of 10 plants from the middle 18 plants in each row.

### Evaluation of cold tolerance at the booting stage under cold water stress

The other two measures of cold tolerance at the booting stage were seed setting rate under cold water (SStR-CW) and relative seed setting rate (RSStR-CW, i.e. ratio of seed setting rate under cold water stress to seed setting rate under normal conditions). Cold tolerance under cold water stress was evaluated in Gongzhuling, Jilin province. Seedlings were grown in a nursery seedbed after planting on 15 April 2006, and transplanted on 25 May with two replications. Twelve plants of each accession were transplanted in a single row with a line × plant distance 25 × 15 cm. The field was fertilized with N (120 kg/ha^2^) and P_2_O_5_ (80 kg/ha^2^). On 1 July at panicle initiation, the field was irrigated continuously for 40 days with cold water pumped from a deep well where the water temperature is a constant 19°C. The cold water was maintained at 20 cm depth at the early stage to 30 cm at the late growth stage, such that the young panicles were covered by cold water. Late accessions that could not be harvested from late August to mid-October were enclosed by a plastic greenhouse to avoid the effects of low atmospheric temperatures on grain filling. Another two replications were transplanted and managed under the same conditions, except that they were irrigated with surface water at higher temperatures (daily mean temperature of 22–26°C from 1 July to 10 August). Mean seed setting rates for all accessions under both cold and normal conditions were recorded on the middle 10 plants in each row.

### SSR polymorphism and genotyping

DNA was extracted from the young leaves using the CTAB method. Two hundred and seventy three simple sequence repeat (SSR) markers randomly distributed across all 12 rice chromosomes (S2 Table) were selected for genotyping. The average distance between markers was 835 kb. The minimum distance between adjacent markers was 0.06 kb and the maximum was 4,050 kb; 18% of distances between adjacent markers were less than 150 kb, more than 50% were less than 750 kb, and more than 90% were less than 1,800 kb. PCR conditions, gel electrophoresis of PCR products and genotype scoring methods were as described in Zhang *et al*. [15].

### Statistical analysis and association mapping

STRUCTURE 2.2 [17], a model-based program, was utilized to infer population structure with 60 unlinked SSRs from the 273 markers. Ten independent simulations were run for each *K* (the number of clusters, from 1 to 10) using a burn-in length of 10,000, run length of 100,000 and a model for admixture and correlated allelic frequencies. To determine the *K* value, the LnP(D) value in the STRUCTURE output and Evanno’s*Δ*K between successive *K* [18] were used.

Pair-wise linkage disequilibria between markers were evaluated by TASSEL V4.0 (http://www.maizegenetics.net/tassel) in a total of 174 accessions and in each of two clusters inferred by STRUCTURE (here, the clusters were subspecies, *indica* and *japonica*).

Association analyses between SSR markers and measures of cold tolerance were carried out by TASSEL V4.0. The Q-Q plot of GLM and MLM for four measures of cold tolerance indicated that MLM overestimated the effect of genetic relationship among individuals (S1 Fig.). The GLM model was therefore used to analyze the association between SSR markers and four measures of cold tolerance for *indica* and *japonica* separately. To control false discovery in association analyses, the significance level ɑ was adjusted upward by ɑʹ in a rate (1-ɑ)^R^ for R-rejected hypotheses. Here, we set ɑ = 0.05, then ɑʹ = 0.05(1–0.05)^R^. We denoted markers with p-values lower than ɑʹ as significantly associated ones.

To provide information about the markers and germplasm resources that may be used in breeding cultivars with strong cold tolerance, we investigated the genotypic effect of each genotype for each QTL. Due to the distinct difference in cold tolerance between the two subspecies, the relative genotypic effect for the i^th^ genotype in the s^th^ subspecies for the corresponding indicator was denoted as $RGE_{\text{si}}=({\bar{x}}_{si}-{\bar{x}}_{s})/{\bar{x}}_{s}$, where, s represents subspecies (*indica* or *japonica*), ${\bar{x}}_{si}$ is the average phenotype of *i* ^*th*^ genotype in the *s* ^*th*^ subspecies, and ${\bar{x}}_{s}$ is the average phenotype of the *s* ^*th*^ subspecies. Error MS for each marker (MS_E_) estimated during association analysis was used to estimate the error MS of RGE as $MS_{RGE\text{si}}=(MS_{E}/n_{\text{i}})/{\bar{x}}_{s}{}^{2}$. Given that MS_E_ was estimated from a large sample, the z-test was used to test whether the RGE of each genotype was biased from zero. We denoted genotypes significantly larger than zero as positive or cold-tolerant genotypes, and those smaller than zero as negative or cold-sensitive genotypes.

## Results

### Population structure and linkage disequilibrium

**Fig 1:**
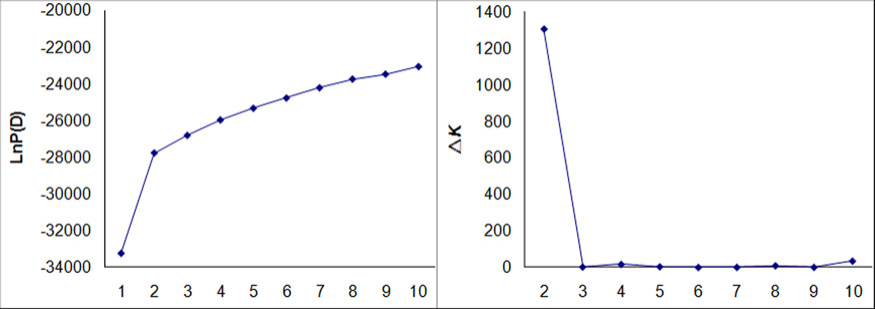
Average LnP(D) and Δ*K* over 10 repeats of STRUCTURE simulation.

**Fig 2:**
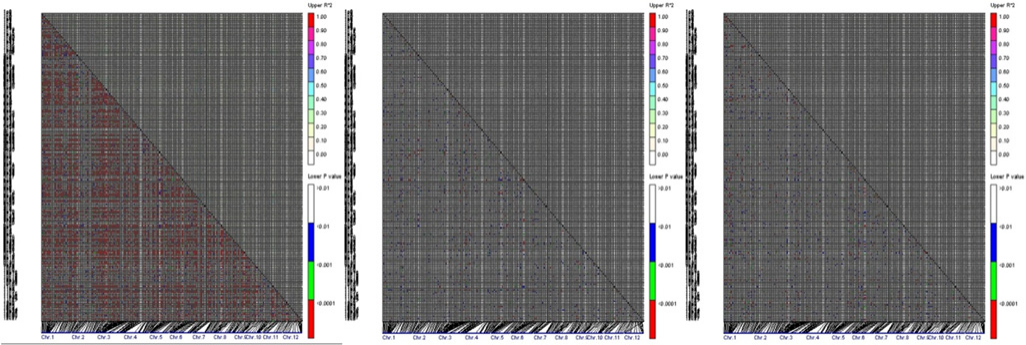
Distribution of LD across 273 SSR loci on 12 linkage groups in the total population (left), *indica* (center) and *japonica* (right).

When we ran the STRUCTURE simulation using 60 SSRs, the LnP(D) value increased with *K* from 1 to 10, but showed an evident knee and there was a sharp peak of Evanno’s Δ*K* at *K* = 2 (Fig. 1). These results indicated that there were two distinctly divergent populations, corresponding to subspecies *indica* and *japonica*. To survey the influence of population structure on LD, we analyzed LD in the whole population and in each of the subgroups (i.e. *indica* and *japonica*) identified by structure analysis (Fig. 2, S3 Table). At the whole population level, the r^2^ within the whole genome was 0.0624±0.0865. For SSR markers with physical distances less than 50 kb, the r^2^ was 0.1443±0.2149; for those between 50 kb and 150 kb, r^2^ was 0.1332±0.1829; for those between 150 kb and 500 kb, r^2^ was 0.0926±0.1353; for those between 500 kb and 1000 kb, r^2^ was 0.0624±0.0765; and for those more than 1,000 kb, r^2^ was 0.0605±0.0818. Thus LD for markers with physical distances shorter than 50 kb was not obviously different from those between 50 kb and 150 kb, but decreased dramatically for those further than 150 kb. Compared to the whole population, LDs within *indica* and *japonica* were obviously lower (S3 Table and Fig. 2). For SSR markers with physical distances shorter than 50 kb, mean r^2^ were 0.1388±0.2135 and 0.1810±0.3203 for *indica* and *japonica*, respectively; for markers between 50 kb and 150 kb, 0.0761±0.0796 and 0.0760±0.0368; for markers between 150 kb and 500 kb, 0.0331±0.0573 and 0.0392±0.0616; for markers between 500 kb and 1000 kb, 0.0155±0.0148 and 0.0336±0.0505; and for markers more than 1000 kb, 0.0134±0.0165 and 0.0201±0.0261. These results indicated that LDs between close markers (such as physical distances less than 50 kb) in both *indica* and *japonica* did not decrease relative to the whole population, however, the LDs between distant markers especially those more than 150 kb decreased dramatically (S3 Table). Given the above results, we carried out the association analysis within *indica* and *japonica* independently in order to avoid false positive associations; and given that few non-linked markers showed strong LD, we used the GLM model that controls population structure but not kinship for the association analysis within *indica* and *japonica*.

### Cold tolerance at the germination and booting stages

All four measures of cold tolerance showed high variation among accessions at both the germination and booting stages, particularly the latter (Table 1). *Japonica* had apparently strong cold tolerance at the germination stage as measured by seedling survival rate (SSvR = 79.11%) under low temperature; this was more than three-fold stronger than *indica* (23.39%). Both *indica* and *japonica* were sensitive to cold stress at the booting stage, as measured by seed setting rate under natural low temperature conditions in Kunming (SStR-NL), seed setting rate under cold water irrigation in Gongzhuling (SStR-CW), and relative seed setting rate under cold water irrigation in Gongzhuling (RSStR-CW). However, *japonica* was more tolerant to cold stress (SStR-NL and RSStR-CW were higher than 25%) than *indica* (SStR-NL and RSStR-CW were lower than 25%). Correlation analyses among the four measures revealed low correlations between cold tolerance at the germination stage and at the booting stage, and low correlations between cold tolerances in different environments (Table 2). Higher correlations in cold tolerance among different stages and environments occurred in the case of *japonica*.

**Table 1:** Phenotypic variation in four cold-tolerant measures in *indica* and *japonica*.

Measure	*Indica*				*Japonica*			
	Mean (%)	Range	SD (%)	CV %	Mean (%)	Range	SD (%)	CV %
SSvR	23.39	1.11–100.00	18.03	77.08	79.11**	1.11–100.00	28.67	36.23
SStR-NL	18.88	0–74.42	21.33	113.01	29.89**	0–80.43	26.41	88.35
SStR-CW	14.71	0–83.49	21.24	144.39	16.87	0–64.31	19.54	115.82
RSStR-CW	14.57	0–89.14	20.53	140.92	25.79*	0–90.47	25.84	100.18

* Significant at P = 0.05;

**: significant at P = 0.01;

SSvR, survival at low temperature expressed as percentage (%) of surviving plants;

SStR-NL, percentage (%) of filled grains per panicle under natural low temperature conditions in Kunming;

SStR-CW, percentage (%) of filled grains per panicle under cold water irrigation in Gongzhuling;

RSStR-CW, percentage (%) relative seed setting under cold water irrigation in Gongzhuling.

**Table 2:** Correlation coefficients among cold-tolerant measures in *indica* (above the diagonal) and *japonica* (below the diagonal).

Measure	SSvR	SStR-NL	SStR-CW	RSStR-CW
SSvR	1.00	0.16	0.11	0.03
SStR-NL	0.16	1.00	0.00	0.17
SStR-CW	0.17	0.23	1.00	0.98 **
RSStR-CW	0.27	0.25	0.91**	1.00

**: Significant at P = 0.01.

### QTLs associated with cold tolerance at the germination and booting stages

**Fig 3:**
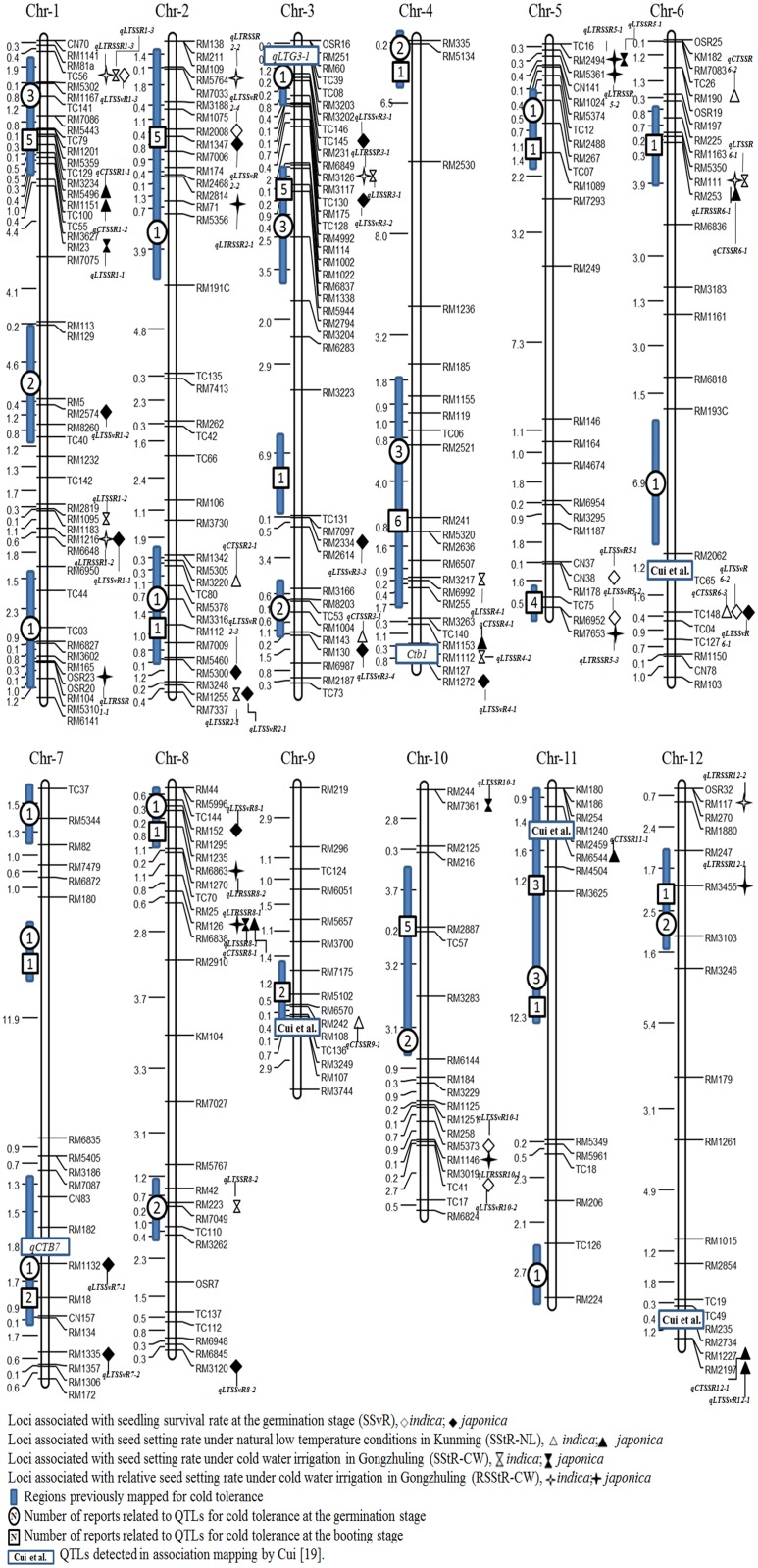
Chromosome maps of QLTs for cold tolerance at the germination and booting stages (the distances between markers are Mb).

Fifty one QTLs related to cold tolerance at the germination and booting stages were detected (S4 Table). They were distributed on all 12 chromosomes, with the maximum number on chromosome 1 and only one on chromosome 9 (Fig. 3). Among them, 22 were detected at the germination stage, 33 at booting, and eight were identified by at least two measures. The genetic contribution of each QTL was 29.20% on average, with a minimum 5.25% (*qLTSSvR3–1* on chromosome 3) and the maximum 59.28% (*qLTSSvR7–1* on chromosome 7). Of the reported QTLs related to cold tolerance in rice (www.gramene.com) more than 54% (21 of 39 QTLs) were detected in the current study (Fig. 3). We identified four loci associated with cold tolerance at the booting stage that were also detected by Cui *et al*. by association mapping [19].

Among the 22 QTLs related to cold tolerance at germination as measured by SSvR, 6 were detected only in *indica* with low average contributions to phenotypic variation (CPV = 12.0%), 15 only in *japonica* with high average CPV (36.2%), and *qCTSSR6–3* in both *indica* and *japonica* with average CPV of 13.5%. The results showing that only one QTL was shared between *indica* and *japonica* and that CPV of QTLs in *japonica* were much higher than those in *indica*, implied that the mechanism of cold tolerance at the germination stage in *japonica* was different from that in *indica*.

Of 12 QTLs associated with SStR-NL, five were detected in *indica* with low average CPV (21.8%), and seven in *japonica* with higher average CPV (33.9%) (Fig. 3, S4 Table). Eight of 12 QTLs associated with SStR-CW were identified in *indica* with low average CPV (24.0%), compared with four in *japonica* with high average CPV (35.2%). Six of 15 QTLs associated with RSStR-CW, were in *indica* with low average CPV (24.3%), compared to nine in *japonica* with high average CPV (38.2%). No QTL related to SStR-NL, SStR-CW or RSStR-CW was shared between *indica* and *japonica*. Under the same stress and environmental conditions, three (*qLTRSSR6–1*, *qLTRSSR3–1* and *qLTRSSR1–3*) of eight QTLs identified by SStR-CW were also detected by RSStR-CW in *indica*, and similar findings were observed for two (*qLTRSSR8–1* and *qLTRSSR5–1*) of four QTLs in *japonica*. Although using the same measure (seed setting rate, SStR), only one QTL (*qLTRSSR8–1*) at the booting stage was detected under both naturally low air temperatures (the corresponding measure is SStR-NL) and cold water irrigation (the corresponding measure is SStR-CW) in *japonica* (Fig. 3). The results indicated that the mechanism of cold tolerance at the booting stage was complex. Moreover, only one QTL (*qLTRSSR8–1*) was detected by the same measure (seed setting rate, SStR) under different kinds of cold stress in *japonica*, i.e. naturally low air temperature in Kunming and cold water irrigation in Gongzhuling.

Among QTLs detected at both the germination and booting stages, the same two QTLs (*qLTSSR1–3* and *qCTSSR6–3* with CPV 9.1 and 10.9%, respectively) were detected at the germination and booting stages in *indica*, but there was no similar QTL in *japonica*. That is, *qLTSSR1–3* and *qCTSSR6–3* may provide a common mechanism of cold tolerance at the germination and booting stages to some degree in *indica*, although most of the cold tolerance at both stages may be attributed to different mechanisms. In *japonica* the mechanisms of cold tolerance at the germination and booting stages appear to be more different than in *indica*.

### Markers and germplasm resources with strong cold tolerance potentially useful for breeding

**Fig 4:**
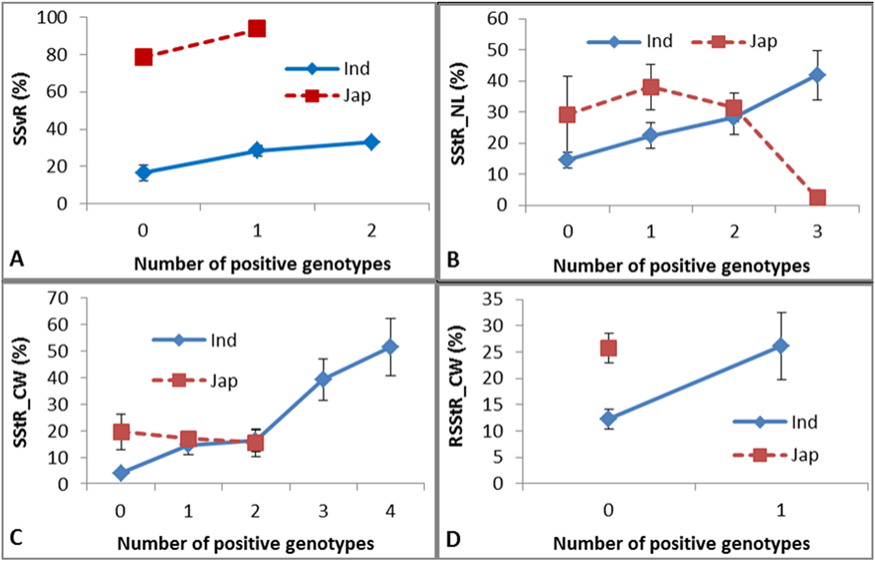
Phenotypes of four measures in *indica* and *japonica* accessions with different numbers of positive QTLs detected in *indica*.

**Fig 5:**
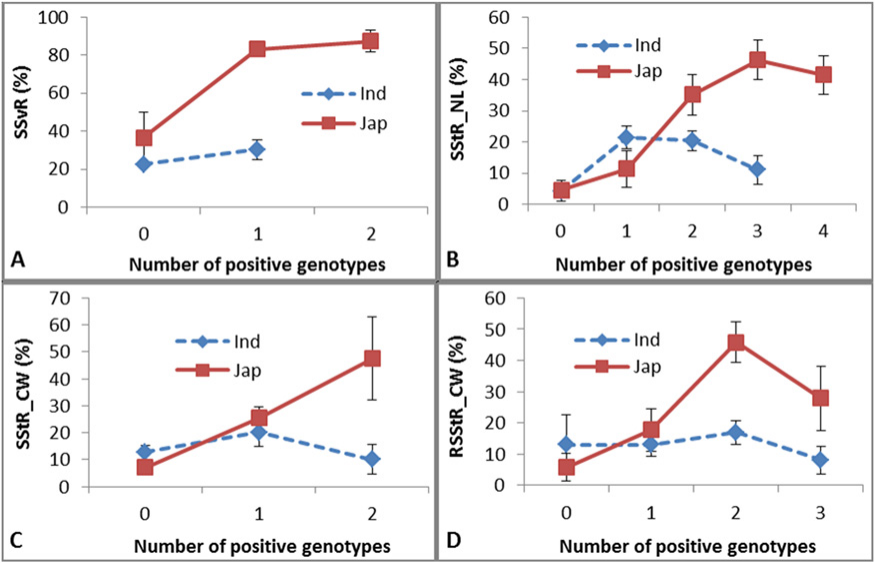
Phenotypes of four measures in *indica* and *japonica* accessions with different numbers of positive QTLs detected in *japonica*.

According to the relative genotypic effect (RGE) of each genotype at each QTL, we detected 18 positive genotypes and 21 negative genotypes in *indica*, and 19 positive genotypes and 24 negative genotypes in *japonica*. Generally, the negative effects were much stronger than the positive effects in both subspecies (S5 Table). Using these genotypes as selection markers we independently screened *indica* and *japonica* accessions containing different numbers of positive genotypes (Fig. 4 and 5). For positive genotypes, the cold tolerance as measured by the four measures increased with the increasing number of positive genotypes in *indica*, but not in *japonica* (Fig. 4). Likewise, for positive genotypes detected in *japonica*, the cold tolerance as measured by the four measures generally increased in *japonica*, but not in *indica*, apart from two exceptions for measures SStR_NL (Fig. 5B) and RSStR_CW (Fig. 5D). We checked the accessions with four genotypes positive for SStR_NL (Fig. 5B), among which one accession (Ye Li Cang Hua) had quite low seed setting (18%) and contained a strong negative genotype (RM5496_136), which reduced seed setting by up to 60% (S5 Table). Similarly, among the accessions with three genotypes positive for RSStR_CW (Fig. 5D), two cultivars (Ning Hui 21 and Dan Dong Lu Dao) had low seed setting (7% and 13.5%, respectively), and both contained a strong negative genotype (RM6863_155) that reduced seed setting by as much as 67% (S5 Table).

In addition to screening accessions using positive genotypes we also screened the top five cultivars showing high cold tolerance according to each measure in *indica* and *japonica*, respectively, and subsequently investigated the distribution of the positive and negative genotypes among these accessions in each subspecies (S5 Table). There were three obvious characteristics for the distribution of the positive and negative genotypes for each measure within each subspecies. Firstly, the top cultivars contained at least one positive genotype of the corresponding measure in the corresponding subspecies, but not necessarily in the other subspecies, although exceptions existed in *indica* for SStR_NL (where accessions Z102, Z146, Z54 and Z64 had no positive genotypes) and RSStR_CW (where accessions Z126, Z5 and Z60 had no positive genotypes). Secondly, the top accessions excluded most of the negative genotypes, especially those with strong negative effects. Thirdly, both positive and negative genotypes in one subspecies may exist in the other subspecies but with no effect on cold tolerance in that subspecies, implying that *indica* and *japonica* do not share the same set of tolerance genes or alleles at most loci.

## Discussion

### QTLs for cold tolerance in *indica* and *japonica*

Association analysis is an effective approach to dissect the genetic bases underlying complex traits in plants [9–10], animals [20] and humans [21]. In the present study we used association analysis to identify 51 QTLs related to cold tolerance at the germination and booting stages in rice. Among them, 46.17% were new QTLs, and 27 corresponded to QTLs reported previously. For example, *qLTSSR4–1* associated with SStR-CW was near the cloned *Ctb1* on chromosome 4; *qLTSSvR7–1* associated with SSvR is 1.4 cM from *qCTB7* on chromosome 7; and *qLTRSSR12–1* associated with RSStR-CW is near *qCTS12*. Four loci identified in the present study were detected at the booting stage by Cui *et al*. [17]. Of these, *qLTSSvR6–1* on Chr6 in the current study is near RM528 as reported by Cui *et al*., *qCTSSR9–1* on Chr9 is near RM160, *qCTSSR11–1* on Chr11 is near RM4B, and *qCTSSR12–1* on Chr12 is near RM235.

Our results clearly indicated that *japonica* has a higher level of tolerance to cold stress than *indica* at both the germination and booting stages. This agrees with results published by others [22–25]. More QTLs were identified in *japonica* (36) than in *indica* (26). Five QTLs were shared among measures in *indica* or *japonica* at the booting stage. Only one QTL (*qCTSSR6–3*) associated with seedling survival rate (SSvR) under low temperatures was shared by *indica* and *japonica*. Reasons for this deserve further discussion.

During the thousands of years of cultivation and utilization under diverse environmental conditions tremendous genetic differentiation has occurred in rice produced under various agro-ecosystems. *Indica* is known to be adapted to tropical environments at low latitudes or altitudes typified by warm climatic conditions, whereas *japonica* cultivars were grown in temperate areas at high latitudes or altitudes with relatively cool conditions [26], at high altitudes in tropical and sub-tropical areas, and in areas where paddy fields are irrigated by cold water. In such areas, water and soil temperatures at sowing are below 15°C [27–28], and thus *japonica* cultivars were preferred due to tolerance to prevailing low temperatures. In double-cropped rice-growing regions, water and soil temperatures at sowing are higher than 15°C but cold air currents from Siberia often occurring in April may cause early rotting of seedlings, resulting in heavy seedling losses and a delayed growing period. *Indica* cultivars grown in such areas are usually characterized by tolerance to short periods of low temperature. Evident correlation has been found between cold stress and altitude or atmospheric temperature. Low temperatures (15–19°C) at booting cause sterile pollen which directly leads to spikelet sterility and ultimately causes serious yield losses [23,29–31]. In Heilongjiang province an increase of 1°C in negative accumulated temperature GDD17 at the inflorescence differentiation stage can result in a 3.51 kg/ha yield loss, and an increase of 1°C in negative accumulated temperature GDD19 at booting stage can cause a 4.95 kg/ha yield loss [32]. *Indica* is adapted to warm areas and is more sensitive to low temperatures. Pollen sterility is observed in *indica* when average temperatures remain below 22°C for three days [33]. The seasonal duration for rice gets progressively shorter due to decreased average temperature and accumulated temperature from southern to northern China. *Japonica* cultivars are mainly grown in the area north of the Yellow River, whereas most *indica* cultivars are grown to the south. This distribution implies that *indica*-*japonica* differentiation resulted from adaptation to different climatic and geographic environments.

The significant *indica*–*japonica* difference in cold tolerance was probably enhanced by rare genetic exchanges between the two subspecies. During cultivation and improvement over thousands of years, *indica* and *japonica* became genetically separated with adaptation to different agro-ecosystems. Differentiation between the subspecies was further enforced by fertility barriers that prevented genetic exchange between the two subspecies [34].

Observations of apparent differences in cold tolerance between *indica* and *japonica* may be partially attributed to the use of SSRs originating from noncoding regions. Only 15% of the SSRs used in the present study were from ESTs: this is higher than the proportion (1.5–7.5%) among all SSRs reported in rice [35–36]. Detection of marker-trait associations based on linkage disequilibria (LD) in genetically diverse materials can help to identify QTLs controlling target traits. However, this depends on the chromosome distance between marker and QTL and times between mutational changes in marker and the causal mutation of the target QTL. An association can be detected easily in a population when the mutation of the test marker is near in distance and time to the functional mutation in the target QTL [37]. However, 85% of SSRs in the study were nonfunctional markers and there are few genomic exchanges between the two subspecies as mentioned above. This may partially explain why *indica* and *japonica* share so few QTLs of cold tolerance.

### Both positive and negative genotypes should be considered in breeding by MAS

Association mapping is a proven and efficient way to detect natural variation in a diverse population and provides many candidate genes or genomic regions for marker assisted selection in breeding. For example, Huang *et al*. used a worldwide panel of cultivated rice varieties to map 32 new loci associated with flowering time and 10 grain-related traits, allowing identification of candidate genes for 18 associated loci [10]. Marker assisted selection is an efficient breeding approach to pyramid elite genes in crop improvement; for example, Narayanan *et al*. developed a line combining blast and blight resistance genes Pi-1, Piz-5 and Xa2 using markers [38]. In the present study, we identified 18 and 19 positive genotypes (i.e. cold-tolerant genotypes) in *indica* and *japonica*, respectively, based on 51 QTLs. Our results indicated that the more positive genotypes the selected cultivars contain, the stronger cold tolerance they show. This proved that positive markers can be used to pyramid cold tolerance genes. In addition to those positive genotypes, however, we identified even more negative genotypes (sensitive to cold stress), numbering 21 and 24 in *indica* and *japonica*, respectively. Some cultivars (such as Ning Hui 21 and Dan Dong Lu Dao) are very sensitive to low temperatures due to a single strong negative genotype (such as RM6863_155) even though they may contain up to three positive genotypes. This indicates that certain strong negative genotypes may play the role of the shortest piece of wood as showed in the bucket theory and should be avoided by the breeders lest they cover up the advantage of the positive genotypes.

## Supporting Information

S1 Fig QQ-plots of GLM and MLM models for four measures of cold tolerance. (DOC)

S1 Table Rice germplasm used in the study. (DOC)

S2 Table SSR markers used in the study. (DOC)

S3 Table LD (r^2^) between markers with different physical distances. (DOC)

S4 Table QTLs based on four measures of cold tolerance. (XLS)

S5 Table Positive and negative genotypes detected in two subspecies and their distribution in the cultivars with top SSvR. (XLS)
