# Supplementary material for: Genetic Analysis of Cold Tolerance at the Germination and Booting Stages in Rice by Association Mapping
Source: PLoS One. 2015 Mar 19;10(3):e0120590. doi: 10.1371/journal.pone.0120590 (PMC4366098; doi:10.1371/journal.pone.0120590)
Supplement: S1 Fig — (DOC) [file pone.0120590.s001.doc]

Fig. S1 QQ-plots of GLM and MLM models for four measures of cold tolerance

GLM for SSvR in *indica* MLM for SSvR in *indica*

GLM for SSvR in *japonica* MLM for SSvR in *japonica*

GLM for SStR-NL in *indica* MLM for SStR-NL in *indica*

GLM for SStR-NL in *japonica* MLM for SStR-NL in *japonica*

GLM for SStR-CW in *indica* MLM for SStR-CW in *indica*

GLM for SStR-CW in *japonica* MLM for SStR-CW in *japonica*

GLM for RSStR-CW in *indica* MLM for RSStR-CW in *indica*

GLM for RSStR-CW in *japonica* MLM for RSStR-CW in *japonica*
