# Supplementary material for: Genetic Analysis of Cold Tolerance at the Germination and Booting Stages in Rice by Association Mapping
Source: PLoS One. 2015 Mar 19;10(3):e0120590. doi: 10.1371/journal.pone.0120590 (PMC4366098; doi:10.1371/journal.pone.0120590)
Supplement: S1 Table — (DOC) [file pone.0120590.s002.doc]

**Table S1 Rice germplasm used in the study**

| **Accession NO.** | **Name** | **Origin** | **Subspecies** | **Landrace or not** |
| --- | --- | --- | --- | --- |
| 10-00463 | Tieganwu | Zhejiang | Japonica | Yes |
| ZD-02715 | Xiangzaoxian7 | Hunan | Indica | No |
| ZD-01001 | Heijing2 | Helongjiang | Japonica | No |
| ZD-01108 | Ergangai | Guangdong | Indica | No |
| ZD-01423 | Xianwanxian1 | Hunan | Indica | No |
| ZD-01820 | Yangdao2 | Jiangsu | Indica | No |
| ZD-02017 | Huke3 | Sichuan | Indica | No |
| ZD-02032 | Aituogu151 | Sichuan | Indica | No |
| ZD-02324 | Liaojing287 | Liaoning | Japonica | No |
| ZD-02495 | Zaoshuxianghei | Guangxi | Indica | No |
| ZD-02547 | Momi | Guangxi | Indica | No |
| ZD-02685 | Jing87-304 | Hunan | Japonica | No |
| ZD-02431 | Huangsiguizhan | Guangdong | Indica | No |
| ZD-02694 | Xianwanxian3 | Hunan | Indica | No |
| ZD-02944 | Zhenxian | Jiangsu | Indica | No |
| ZD-03104 | Zaoxian | Anhui | Indica | No |
| ZD-03115 | Dangyu5 | Anhui | Indica | No |
| ZD-03525 | Zhengdao5 | Henan | Japonica | No |
| ZD-03867 | Sibeitizhao6 | Beijing | Japonica | No |
| 16-05252 | Hongainuo | Guangxi | Indica | Yes |
| 18-04906 | Wanlixian | Hunan | Indica | Yes |
| 22-04637 | Xiaobaimi | Guizhou | Indica | Yes |
| 13-01433 | Yanshuichi | Fujian | Indica | Yes |
| ZD-02605 | Jinyou1 | Fujian | Indica | No |
| 15-04286 | Xishi | Guangdong | Japonica | Yes |
| 16-02459 | Hongjinghangu | Guangxi | Indica | Yes |
| 21-02089 | Lamujia | Yunnan | Japonica | Yes |
| 21-02235 | Yuyannuo | Yunnan | Japonica | Yes |
| A0434 | 80B | Hunan | Indica | No |
| R0004 | Gu154 | Hunan | Indica | No |
| R0014 | Gui630 | Hunan | Indica | No |
| R0032 | IR661-1 | Hunan | Indica | No |
| R0215 | PeiC122 | Hunan | Japonica | No |
| R0333 | Jing7623 | Shanghai | Japonica | No |
| ZD-03386 | Chengnongshuijing | Sichuan | Indica | No |
| R0337 | Ninghui21 | Jiangsu | Japonica | No |
| R0430 | Jan-76 | Liaoning | Japonica | No |
| R0447 | Huhui628 | Hunan | Japonica | No |
| R0468 | Tingxuanhui | Hunan | Indica | No |
| R0515 | Xianghui91269 | Hunan | Indica | No |
| R0604 | JWR221 | Jiangsu | Indica | No |
| 16-00163 | Hengxianliangchun | Guangxi | Indica | Yes |
| 18-01067 | Baikehanhe | Hunan | Indica | Yes |
| 19-00022 | Xiangdao | Henan | Indica | Yes |
| 20-03215 | Shanjiugu | Sichuan | Japonica | Yes |
| 16-09350 | Guangkexiangnuo | Guangxi | Japonica | Yes |
| 21-01744 | Laozaogu | Yunnan | Indica | Yes |
| 21-01989 | Lengshuinuo | Yunnan | Japonica | Yes |
| 21-02851 | Haoxiang | Yunnan | Indica | Yes |
| 22-04574 | Haoluguangzhan | Guizhou | Japonica | Yes |
| A0016 | JinnanteB | Hunan | Indica | No |
| A0060 | ZhuzhenB | Hunan | Indica | No |
| A0086 | Zhaoyangyi1B | Hunan | Indica | No |
| A0096 | L 301B | Hunan | Indica | No |
| A0112 | AnnongwanjingB | Hunan | Japonica | No |
| A0132 | Zaoshunonghu6 | Hunan | Japonica | No |
| A0430 | Baoxie123B | Hunan | Indica | No |
| A0172 | Qingsiai16B | Guangdong | Indica | No |
| A0244 | XiangaiB | Jiangxi | Indica | No |
| A0246 | Jiangnongzao1 | Jiangxi | Indica | No |
| A0298 | JinghuB | Anhui | Japonica | No |
| A0408 | Zhenrui409B | Yunnan | Indica | No |
| A0464 | Baoxie-7B | Hunan | Indica | No |
| A0596 | Gzhenxian97B | Sichuan | Indica | No |
| A0598 | 88B | Jiangsu | Indica | No |
| 11-00403 | Leihuozhan | Anhui | Indica | Yes |
| 30-00206 | Taizhong65 | Taiwan | Japonica | Yes |
| 21-04506 | Piwusheng | Yunnan | Indica | Yes |
| 30-00210 | Taizhongzailai1 | Taiwan | Indica | Yes |
| 20-01452 | Mamagu | Sichuan | Indica | Yes |
| 20-02073 | Meihuanuo | Sichuan | Indica | Yes |
| 12-02373 | Aimi | Jiangxi | Indica | Yes |
| 02-00295 | Yelicanghua | Hebei | Japonica | Yes |
| 05-00024 | Weiguo | Liaoning | Japonica | Yes |
| 09-00530 | Baigedao | Jiangsu | Japonica | Yes |
| 09-00724 | Huangkezaonian | Jiangsu | Japonica | Yes |
| 11-00322 | Liushizao | Anhui | Indica | Yes |
| 21-00083 | Haomake(K) | Yunnan | Japonica | Yes |
| 02-00058 | Funingzipi | Hebei | Japonica | Yes |
| 21-01257 | Qingke | Yunnan | Indica | Yes |
| 21-02852 | Haohuangla | Yunnan | Indica | Yes |
| 21-03121 | Nangaogu | Yunnan | Indica | Yes |
| 21-05048 | Ximaxian | Yunnan | Japonica | Yes |
| 07-00010 | Laoguangtou83 | Helongjiang | Japonica | Yes |
| 05-00052 | Dandongludao | Liaoning | Japonica | Yes |
| 07-00109 | Baimaodao | Helongjiang | Japonica | Yes |
| ZD-01559 | Xiushui115 | Zhejiang | Japonica | No |
| 08-00036 | Muxiqiu | Shanghai | Japonica | Yes |
| 08-00066 | Laohuzhong | Shanghai | Japonica | Yes |
| 09-01361 | Cunsanli | Jiangsu | Japonica | Yes |
| 11-00389 | Qiuqianbai | Anhui | Indica | Yes |
| 11-00529 | Feidongtangdao | Anhui | Japonica | Yes |
| 12-00589 | Jinxibai | Jiangxi | Indica | Yes |
| 12-02254 | Taishannuo | Jiangxi | Indica | Yes |
| 12-02280 | Aihechi | Jiangxi | Indica | Yes |
| 13-00723 | Jinbaoyin | Fujian | Indica | Yes |
| 13-00737 | Minbeiwanxian | Fujian | Indica | Yes |
| ZD-00560 | Nanjing11 | Jiangsu | Indica | No |
| 13-00816 | Lucaihao | Fujian | Indica | Yes |
| 13-01301 | Yizhixiang | Fujian | Indica | Yes |
| 15-00503 | Shuyazhan | Guangdong | Indica | Yes |
| 15-00648 | Simiao | Guangdong | Indica | Yes |
| 15-03025 | Qimei | Guangdong | Indica | Yes |
| 15-03057 | Nanxiongzaoyou | Guangdong | Indica | Yes |
| 15-03336 | Heidu4 | Guangdong | Indica | Yes |
| 15-03586 | Cikenuo | Guangdong | Japonica | Yes |
| 15-04016 | Sanlicun | Guangdong | Indica | Yes |
| 16-06887 | Qiyuexian | Guangxi | Indica | Yes |
| ZD-00002 | Guangluai4 | Guangdong | Indica | No |
| 17-00502 | Dongtingwanxian | Hubei | Indica | Yes |
| 17-00524 | Liuyezhan | Hubei | Indica | Yes |
| 17-00966 | Xuanenchangtan | Hubei | Indica | Yes |
| 17-01470 | Bawangbian1 | Hubei | Indica | Yes |
| 18-01903 | Xugunuo | Hunan | Indica | Yes |
| 18-03950 | Muguanuo | Hunan | Japonica | Yes |
| 18-04082 | Hongqi5 | Hunan | Japonica | Yes |
| 19-00205 | Hanmadao | Henan | Indica | Yes |
| 20-01262 | Xibaizhan | Sichuan | Indica | Yes |
| 20-01734 | Nantiangangjiu | Sichuan | Japonica | Yes |
| ZD-01006 | Guichao2 | Guangdong | Indica | No |
| 20-02821 | Zhongnong4 | Sichuan | Indica | Yes |
| 20-03042 | Honggu | Sichuan | Japonica | Yes |
| 20-03053 | Sankecun | Sichuan | Indica | Yes |
| 21-00529 | Sanpangqishiluo | Yunnan | Japonica | Yes |
| 21-00694 | Qitoubaigu | Yunnan | Indica | Yes |
| 21-00785 | Benbanggu | Yunnan | Japonica | Yes |
| 21-01106 | Zimi | Yunnan | Indica | Yes |
| 21-01165 | Xiaohonggu | Yunnan | Indica | Yes |
| 21-01577 | Wuzigu | Yunnan | Japonica | Yes |
| 21-01899 | Gongju73 | Yunnan | Indica | Yes |
| 30-00195 | Taidongludao | Taiwan | Japonica | Yes |
| 21-02171 | Qitougu | Yunnan | Indica | Yes |
| 21-02224 | Zinuo | Yunnan | Indica | Yes |
| 21-02769 | Mowanggunei | Yunnan | Indica | Yes |
| 21-02824 | Haolai | Yunnan | Indica | Yes |
| 21-03433 | Jinzhinuo | Yunnan | Indica | Yes |
| 21-03781 | Jixuenuo | Yunnan | Japonica | Yes |
| 21-03879 | Fanhaopi | Yunnan | Indica | Yes |
| 21-05072 | Wuzuihonggu | Yunnan | Indica | Yes |
| 21-05171 | Beizinuo | Yunnan | Indica | Yes |
| 22-00040 | Zhegu | Guizhou | Indica | Yes |
| 30-00244 | Taizhongxianxuan2 | Taiwan | Indica | Yes |
| 22-00513 | Magunuo | Guizhou | Japonica | Yes |
| 22-00570 | Xiangnuo | Guizhou | Japonica | Yes |
| 22-01439 | Niankenuo | Guizhou | Japonica | Yes |
| 22-01615 | Maweizhan | Guizhou | Indica | Yes |
| 22-01843 | Hongkezhenuo | Guizhou | Japonica | Yes |
| 22-02148 | Cungunuo | Guizhou | Japonica | Yes |
| 22-02754 | Youzhan | Guizhou | Indica | Yes |
| 22-03815 | Guantuibaihe1 | Guizhou | Japonica | Yes |
| 22-04053 | Yangkenuo | Guizhou | Japonica | Yes |
| 24-00195 | Maguzi | Shanxi | Japonica | Yes |
| 12-00644 | Jiefangxian | Jiangxi | Indica | Yes |
| 24-00215 | Laohongdao | Shanxi | Japonica | Yes |
| 26-00008 | Jiabala | Xizang | Indica | Yes |
| 28-00005 | Heimangdao | Ningxia | Japonica | Yes |
| 29-00010 | Putaohuang | Tianjing | Japonica | Yes |
| 31-00032 | Menjiagao1 | Hainan | Indica | Yes |
| ZD-01195 | Baoxuan21 | Guangdong | Indica | No |
| 21-00272 | Wenxiangnuo | Yunnan | Indica | Yes |
| 21-00357 | Haobuka | Yunnan | Japonica | Yes |
| 21-01082 | Dawannuo | Yunnan | Indica | Yes |
| 21-01120 | Xianggu | Yunnan | Indica | Yes |
| 12-02850 | Hongmisandanbai | Jiangxi | Japonica | Yes |
| 21-01853 | Haonayong1 | Yunnan | Japonica | Yes |
| 21-01970 | Lengshuigu2 | Yunnan | Japonica | Yes |
| 31-00042 | Menjiading2 | Hainan | Indica | Yes |
| 21-02619 | Huangpinuo | Yunnan | Japonica | Yes |
| 22-02356 | Feienuo | Guizhou | Japonica | Yes |
| 17-00435 | Dongtingwanxian | Hubei | Indica | Yes |
| ZD-00358 | Binwan3 | Hunan | Indica | No |
| ZD-00747 | Aimakang | Sichuan | Indica | No |
| ZD-00760 | Shufeng101 | Sichuan | Indica | No |
| ZD-00806 | Lixinjing | Sichuan | Japonica | No |
