# Supplementary material for: Genetic Analysis of Cold Tolerance at the Germination and Booting Stages in Rice by Association Mapping
Source: PLoS One. 2015 Mar 19;10(3):e0120590. doi: 10.1371/journal.pone.0120590 (PMC4366098; doi:10.1371/journal.pone.0120590)
Supplement: S2 Table — (DOC) [file pone.0120590.s003.doc]

**Table S2 SSR markers used in the study**

| **Locus** | **Chr.** | **Forward primer** | **Reverse primer** |
| --- | --- | --- | --- |
| OSR23 | 1 | tgatacgtggtacgtgacgc | taatcgcttccctacccctg |
| RM104 | 1 | ggaagaggagagaaagatgtgtgtcg | tcaacagacacaccgccaccgc |
| RM1095 | 1 | cccattcagttgatcctgtc | gcaaaagcaaggatggagac |
| RM113 | 1 | caccattgcccatcagcacaac | tcgccctctgctgcttgatggc |
| RM1141 | 1 | tgcattgcagagagctcttg | cagggctttgtaagaggtgc |
| RM1151 | 1 | gaccgcaaaagatcatcgac | gtaacagcgaccgttggttg |
| RM1183 | 1 | gggcacgaataaaaccagag | gggatggtccaatgacaaag |
| RM1201 | 1 | ttaccgcgccacatatacac | cgtacgagccctagttaccg |
| RM1216 | 1 | ttccccaatggaacagtgac | agggtctaccacccgatctc |
| RM1232 | 1 | gtctctgtggagtggaagcc | ttcaccggatctgattaccc |
| RM129 | 1 | tctctccggagccaaggcgagg | cgagccacgacgcgatgtaccc |
| RM165 | 1 | ccgaacgcctagaagcgcgtcc | cggcgaggtttgctaatggcgg |
| RM23 | 1 | cattggagtggaggctgg | gtcaggcttctgccattctc |
| RM2574 | 1 | cttgggttcgagtaggataa | tccaccagaatttgatcaat |
| RM3234 | 1 | aaagacgacgatgggtcaac | gtgaggttcttgggtggaag |
| RM3627 | 1 | ggctactcgagcaagctctg | acctacccgtcatccctctc |
| RM5 | 1 | tgcaacttctagctgctcga | gcatccgatcttgatggg |
| RM5302 | 1 | tatgggtgacacattgggac | ttgtgacgtttgagagctgg |
| RM5310 | 1 | tagacaaagcaacgggttcc | cggaagcaggagaatcgtag |
| RM5359 | 1 | cgtgatctcgtgcatccc | ccctcaggagcttcatgaac |
| RM5443 | 1 | tacggcttacccatagcagc | aaacggagggagtatttccc |
| RM5496 | 1 | tgcctactcagcaactaacac | actttgcagtttgcacatc |
| RM6827 | 1 | gcaccgaacaaaatcctagc | ccaacctcaactgaagatgc |
| RM6950 | 1 | gtctgtgtcactaaccatgcc | catggcgtctcaactacacc |
| RM7075 | 1 | tatggactggagcaaacctc | ggcacagcaccaatgtctc |
| RM7086 | 1 | tattttgcctccaagaggcc | ggtgcatggttctgaggaac |
| RM81a | 1 | gagtgcttgtgcaagatcca | cttcttcactcatgcagttc |
| RM8260 | 1 | aatctaacgtttgactatccatc | tctaccagtactcccttcacc |
| TC03 | 1 | ccaaggcagcgcttgttc | tggtggagttctcgatcctc |
| TC100 | 1 | ttctcttctcctacctagcaacc | acaagccatacccataccca |
| TC129 | 1 | cagacaagcagcaagcagtc | cgaccacaaaagcctaccat |
| TC141 | 1 | tagtcgccttgattggctct | tccagatcctttggttcctg |
| TC142 | 1 | atgaaccgaactaccgaacg | tcgcattgctggtatgtgtt |
| TC44 | 1 | ttctacgacttcttcggcgt | agtttcaaaaagcacgacgg |
| TC55 | 1 | aaaccgaccaaaatcactgc | acgccaaatccatctctcaa |
| TC56 | 1 | gccgctataaatcgaaccaa | gtatgtacccaccaccaggg |
| TC79 | 1 | gccaccaaacgaacacct | aagcctcctcctcattccac |
| RM106 | 2 | cgtcttcatcatcgtcgccccg | ggcccatcccgtcgtggatctc |
| RM109 | 2 | gccgccggagagggagagagag | ccccgacgggatctccatcgtc |
| RM1251 | 2 | gagacaatgacagtctgcgc | ccttcagcccttcacgtatc |
| RM1255 | 2 | catctgcttctgctaagctagg | cccaataagcagctaagctc |
| RM1342 | 2 | gaagcaagaaaccaaagatg | ctttcggtctcaagcaatat |
| RM1347 | 2 | aacaaattaaactgccaag | gtcttatcatcagaactgga |
| RM138 | 2 | agcgacgccaagacaagtcggg | tccacgtcgatcgacacgacgg |
| RM191C | 2 | cccatcctcaccgatctctctaaac | gtgcgcacggaggaggaaaggg |
| RM211 | 2 | ccgatctcatcaaccaactg | cttcacgaccatctcaaagg |
| RM2468 | 2 | tcccctgcctctaattaatc | aagtcaaagtgtcaagaccaaa |
| RM262 | 2 | cattccgtctcggctcaact | cagagcaaggtggcttgc |
| RM3188 | 2 | tcacgagtcgttcgttcttg | cttgctgctcaagtggtgag |
| RM3220 | 2 | ttgagttttcctggccagtc | ctcgctttacaggccagaac |
| RM3248 | 2 | agaaggttgctttcttggcc | cttgcaaggtctgttgcatc |
| RM3316 | 2 | ttcgacgattctgtacacgc | catgatcccaaatgcatggg |
| RM3730 | 2 | tgcgagtatcttcaaggcag | attgagggggctaatcatcc |
| RM5300 | 2 | ccaccccatcattattgagg | aagctgaggttggttgcttg |
| RM5305 | 2 | ccttccctatgctatgctgc | gatggggagtaatggtgtgg |
| RM5378 | 2 | gctcggctgcgttctactac | agaaaggagggagccgatag |
| RM5460 | 2 | aagagaacaagccatggtgc | gccttttcttgcctttggac |
| RM5764 | 2 | cgacgctgtctcttgttgag | cattcgtttcaccaatggcc |
| RM7006 | 2 | ctcgtttatcctcccagtgc | cacttgtatccagaagcagg |
| RM7009 | 2 | gggatttattggtcggactg | gtaaggcggcacaaagaatc |
| RM7033 | 2 | gtgcccaacactgcactaac | gttggcggtgatttctgatg |
| RM71 | 2 | ctagaggcgaaaacgagatg | gggtgggcgaggtaataatg |
| RM7337 | 2 | ttcttcccagttgggttgac | catcttgttgatggtggtgg |
| RM7413 | 2 | gtctggttggcagctctctc | cgacacacatccacgcac |
| TC135 | 2 | agccaagccaagaagacaaa | aacatcccaactcgaacacc |
| TC42 | 2 | tagatcgaatcgacagtgcg | gagaaataacgcgtgccatc |
| TC66 | 2 | ctgtgtagcagaagcatggc | tccatccccaagaatacagc |
| TC80  RM1002 | 2  3 | aagttgcgattggtgaaagg  gaaccagacaagcaaaacgg | gttgctgacatgccatagga  agcatggggatttaggaacc |
| RM1004 | 3 | acgacccctcctggttctg | ctcgtggttctggtcacaac |
| RM1022 | 3 | catgggatgagggagtaatg | ctttgatagcggctttgtcc |
| RM114 | 3 | cagggacgaatcgtcgccggag | ttggcccccttgaggttgtcgg |
| RM130 | 3 | tgttgcttgccctcacgcgaag | ggtcgcgtgcttggtttggttc |
| RM1338 | 3 | agagggaattagattggatt | ggtccacttcttccttctat |
| RM143 | 3 | gtcccgaaccctagcccgaggg | agaggccctccacatggcgacc |
| RM175 | 3 | cttcggcgccgtcatcaaggtg | cgttgagcagcgcgacgttgac |
| RM2187 | 3 | gtcatttgaagtaaatccgt | ggtctacttgcgaaataagt |
| RM231 | 3 | ccagattatttcctgaggtc | cacttgcatagttctgcattg |
| RM2334 | 3 | catgcatctgatctgattat | tgtgaagagtacaagtaggg |
| RM251 | 3 | gaatggcaatggcgctag | atgcggttcaagattcgatc |
| RM2614 | 3 | tggcacaaatcattatgatc | gattgcaatgcagcatatag |
| RM3117 | 3 | gccatctctctctctctctctc | ccttagctcatcaagcgagg |
| RM3126 | 3 | ttcttgctcgtctgcctcc | catcttgccatgcctgatg |
| RM3166 | 3 | aaatcgtcgaacacctctcg | ttcacacgcatcgagtaagc |
| RM3202 | 3 | ttcacttcctattggcggc | tcatcatcagtccagcatcg |
| RM3203 | 3 | agagcatcatgcaggtcctc | atacgaatggagtgcaaggg |
| RM3204 | 3 | gcaaccctttcttcctcctc | ccaaggagagcgcactagc |
| RM3223 | 3 | agagcatcatgcaggtcctc | atacgaatggagtgcaaggg |
| RM4992 | 3 | cagcctgctaatttagtatt | actcgaaatccttctctata |
| RM5944 | 3 | gagccgcatcaaccagttac | cagtacagcgcgcactacac |
| RM60 | 3 | agtcccatgttccacttccg | atggctactgcctgtactac |
| RM6283 | 3 | tggagactgagctgatgcc | tcaggtggtcggttccttac |
| RM6837 | 3 | acctggtgcaagaacctgac | cggtagaggacgtccatgtc |
| RM6849 | 3 | cgtcaactgcatcaccacc | tccgactgatcatcatcgac |
| RM6987 | 3 | cgatccttaccttgaattga | gaaagccattcagtgaactg |
| RM7097 | 3 | gggaggaggagaggagattg | ttaggcctgcacttttggag |
| RM8203 | 3 | cattgataatgtccagtgacg | ctcctgttgtcattctttgg |
| TC08 | 3 | cctgacctcactgcacttca | agcttggcttgattgctgat |
| TC128 | 3 | agaggaagggcaaggagaag | cagcaaaaaccaccgagatt |
| TC130 | 3 | ttccccaagtacacgagagg | tactgctgcccgcttctaat |
| TC145 | 3 | caagtcctaacccgaatcca | gggcttgacctcaagtagca |
| TC146 | 3 | ttggatttgtgcttgtgctc | tcagtagtctgccaaatcttgaa |
| TC39 | 3 | atctcatcgacccatccaaa | tgacttgaggttgaggagca |
| TC53 | 3 | gatgcttagccatcatccgt | cgagtcgatcctcacacaaa |
| TC73 | 3 | actacgtcaggctcgtcgtc | agtttactcggcagcaacca |
| RM1112 | 4 | tcaggacacatggcccttac | cagctcctgacagagcacac |
| RM1153 | 4 | accaacgccaaaagctactg | tactcgccctgcatgagc |
| RM1155 | 4 | agggagtgtggcaactatgc | gggaggagtgagaagggatc |
| RM119 | 4 | catccccctgctgctgctgctg | cgccggatgtgtgggactagcg |
| RM127 | 4 | gtgggatagctgcgtcgcgtcg | aggccagggtgttggcatgctg |
| RM1272 | 4 | tctatggatctgcatgctgg | ctgccctgtccttttaatcg |
| RM241 | 4 | gagccaaataagatcgctga | tgcaagcagcagatttaggtg |
| RM255 | 4 | tgttgcgtgtggagatgtg | cgaaaccgctcagttcaac |
| RM2636 | 4 | cggaggaagtaccttataaa | cttctcagattcttgtgtgt |
| RM3217 | 4 | gttgcaaggttgcaacacag | gtggcagccaagatggac |
| RM3263 | 4 | ccccctcctttaatttgcac | ctcctgatcctcatggatgg |
| RM335 | 4 | gtacacacccacatcgagaag | gctctatgcgagtatccatgg |
| RM5134 | 4 | gattggagcttgttttctc | cacaaatcaaatacatcacag |
| RM5320 | 4 | cctgagctgtacaagcaaac | cagattcttgggagaaatcc |
| RM6507 | 4 | cggatgattcgtatgtgcag | aacacgatgttggcaaggac |
| RM6992 | 4 | attacctgctttcccactgc | ctcacgtgtactgccaatcc |
| TC06 | 4 | gtatgatgtgcccggctatt | tcgaactttatcccctttcag |
| TC140 | 4 | ttgcattggctcaatgatgt | tttcccttcttccttcctcc |
| CN157 | 5 | aaggccgcgagaggattc | catcgtgaacgccatctct |
| CN38 | 5 | cctgctcgcgtgaaagata | cctcctcgatctggatggt |
| CN55 | 5 | gctgatagcgaggtgggtag | ctgccggttgatcttgttct |
| CN70 | 5 | ctgcctcgcgtgaaagata | cctcctcgatctggatggt |
| CN72 | 5 | gcttgggtgatttcttggaa | ggccgaagaggcggtagatctt |
| RM1024 | 5 | gcatataccatggggattgg | gggattgggataatggtgtg |
| RM1089 | 5 | cagaaggattatctcgatacc | aatagggcttgaaataaattg |
| RM1187 | 5 | gtggctatggctactgagcc | ccgttgttggtatccaggtc |
| RM146 | 5 | ctattattccctaacccccataccctcc | agagccactgcctgcaaggccc |
| RM164 | 5 | tcttgcccgtcactgcagatatcc | gcagccctaatgctacaattcttc |
| RM178 | 5 | tcgcgtgaaagataagcggcgc | gatcaccgttccctccgcctg |
| RM249 | 5 | ggcgtaaaggttttgcatgt | atgatgccatgaaggtcagc |
| RM2494 | 5 | ggattaatgaaatggaacac | cataccagtgcaaaacatag |
| RM267 | 5 | tgcagacatagagaaggaagtg | agcaacagcacaacttgatg |
| RM3295 | 5 | tcgtgtcatgcgatcgac | gcttcgactcgaccaagatc |
| RM4674 | 5 | agcattatccatattcacat | taatcggacataagactttc |
| RM5361 | 5 | gcacgtgactccatcatctc | atgcagatgatagcccaagg |
| RM5374 | 5 | catgatgaatgtattgctct | acatggtcaaccattttaat |
| RM6952 | 5 | actccatgacggaatcgaac | ggacatcaaaggcaccattc |
| RM6954 | 5 | cacagatgcgaaatgcagag | gcgctgctgctaaattaagc |
| RM7293 | 5 | cctaggggatccaagatgtc | gcacggatctacatacatgc |
| RM7653 | 5 | aattcgtccccgtctcctac | gaattccagctctttgaccg |
| TC07 | 5 | ggctgagattccttccttcc | taacaaaacttctcggcgct |
| TC12 | 5 | cttggaaagctggaacttgg | cccacctcacctcacctcta |
| TC16 | 5 | gccaagaagaaccaggagc | tgcttggtggatgaattgaa |
| TC75 | 5 | gaatcgaatgctgctgtcaa | cacaggtcagaaaacgagca |
| CN78 | 6 | gcaagtgggcgctctcct | gtccatgagcctggacacctc |
| RM1015 | 6 | tgtatgactttttagcattg | ccacattcatttagatgtta |
| RM103 | 6 | cttccaattcaggccggctggc | cgccacagctgaccatgcatgc |
| RM111 | 6 | cacaacctttgagcaccgggtc | acgcctgcagcttgatcaccgg |
| RM1150 | 6 | acagtggccacagtgtgttg | ggattcgggaggttgacg |
| RM1161 | 6 | aaactgttttacccctggcc | atccccttctgcggtaaaac |
| RM1163 | 6 | tctagggttagggtttcgcc | aggtcggtttccttttgtcc |
| RM190 | 6 | ctttgtctatctcaagacac | ttgcagatgttcttcctgatg |
| RM193 | 6 | cgcctcttcttcctcgcctccg | cgggtccatcccccctctcctc |
| RM197 | 6 | gatccgtttttgctgtgccc | cctcctctccgccgatcctg |
| RM2008 | 6 | atagttgaagcattttccag | aacccatggagaatgtatag |
| RM2062 | 6 | attttgacttcttgtttcta | ctttcaagttgttaagtttg |
| RM225 | 6 | tgcccatatggtctggatg | gaaagtggatcaggaaggc |
| RM253 | 6 | tccttcaagagtgcaaaacc | gcattgtcatgtcgaagcc |
| RM3183 | 6 | gctccacagaaaagcaaagc | tgcaacagtagctgtagccg |
| RM5350 | 6 | agtttggactgcccaatcag | ttggagggggatgaatgtc |
| RM6818 | 6 | gtcgcattcgtctccacc | accatttccagatgactcgg |
| RM6836 | 6 | ttgttgtatacctcatcgac | agggtaagacgtttaacttg |
| RM7083 | 6 | tgtgttttggtgtgcctgac | actaccgtggtaccaaacgg |
| TC04 | 6 | actgaaaggaggcagaagca | ggcaatcttgacacagctca |
| TC127 | 6 | gaatccaagtccagggttga | cactctcgaggagatgggtt |
| TC148 | 6 | acaaatcaacagccaccaca | agccatttggaacagattgg |
| TC26 | 6 | cgtgtcgcttactctagggc | cctgtaagggagcacgtcat |
| TC65 | 6 | ggagaacaagaagctgaccg | gctgtcgacgctacggat |
| CN83 | 7 | atcgacggcacgatcaag | ggtggcagtggaagtgctat |
| RM1132 | 7 | atcacctgagaaacatccgg | ctcctcccacgtcaaggtc |
| RM1335 | 7 | gcatgcatgaatatgatgg | agatcgaacaagaagagtgg |
| RM134 | 7 | acaaggccgcgagaggattccg | gctctccggtggctccgattgg |
| RM172 | 7 | tgcagctgcgccacagccatag | caaccacgacaccgccgtgttg |
| RM18 | 7 | ttccctctcatgagctccat | gagtgcctggcgctgtac |
| RM182 | 7 | tgggatgcagagtgcagttggc | cgcaggcacggtgccttgtaag |
| RM3186 | 7 | gagtagaaggtgaggccacg | cgaccaagagatgcttcctc |
| RM5344 | 7 | acgaacgggagcaaggtc | ctctcaaccaagacgccttc |
| RM5405 | 7 | cactctcacactcaccagcg | gtcgtctcgctctcatctcc |
| RM6835 | 7 | ttctgctccacgtgttcttg | taacccatagtcccgtacgc |
| RM6872 | 7 | ggatgaacactgatgatggc | acctccaccacgatatccac |
| RM7087 | 7 | agctagcagctattgcctgc | cagtgagtgagtgcactggc |
| RM7479 | 7 | gctctggttagtgatcatgg | acatggtggcttaggagtg |
| RM82 | 7 | tgcttcttgtcaattcgcc | cgactcgtggaggtacgg |
| TC37 | 7 | acaacagccaccaactcctc | cgttcagctcctcgtagtcc |
| RM1235 | 8 | gaaaactaaaaagcagagga | aagctatccattttggatta |
| RM126 | 8 | cgcgtccgcgataaacacaggg | tcgcacaggtgaggccatgtcg |
| RM1270 | 8 | tactagttcactaccacgcagc | gcatttcccgcaatgtagag |
| RM1295 | 8 | gagaagaggtggaagttgaa | gacggaagaattcttaatgg |
| RM152 | 8 | gaaaccaccacacctcaccg | ccgtagaccttcttgaagtag |
| RM223 | 8 | gagtgagcttgggctgaaac | gaaggcaagtcttggcactg |
| RM25 | 8 | ggaaagaatgatcttttcatgg | ctaccatcaaaaccaatgttc |
| RM2819 | 8 | aatgttgctagatttaaaac | cagtaggatatcttacaacc |
| RM2910 | 8 | cagctgctcatattcatata | ataaggtacttcatccgtta |
| RM3120 | 8 | atcgatggaagctctttgcc | ggatgtacaagagcttaggagc |
| RM3262 | 8 | accgatgagctctccacatc | tgacctcacttcacttcccc |
| RM42 | 8 | atcctaccgctgaccatgag | tttggtctacgtggcgtaca |
| RM5767 | 8 | ctagcagccacatcaagcag | ctcatcctctccacgctctc |
| RM6838 | 8 | attaataccgctaccacgcg | tcctcctccacctcaatcac |
| RM6845 | 8 | gtgacggcaagaggaagaag | gttcgacaggaacgccac |
| RM6863 | 8 | gctgcagaattaaggagaac | tgctcaaaataatcagctcc |
| RM6948 | 8 | ggtaagttgtcggttgcctc | acgtccataccaggtcaagc |
| RM7027 | 8 | aggacctggactttatgggc | cctgcactgctccacagtac |
| RM7049 | 8 | ctaatccgtggatcaaacgc | gttgagcaaacgtctgttgg |
| TC110 | 8 | cgatcgaggagttcgtcttc | caccccttcacatgatcaca |
| TC112 | 8 | caagctagtcacccacccat | agcacatcacaaaccaacca |
| TC137 | 8 | acctccgccttcttaacctc | gacatggctggtttcttggt |
| TC144 | 8 | tcttcttgcagacgacgaga | ccattgacgacacacacaca |
| TC70 | 8 | caaaaaggaggtgaaggcaa | actgcattggatgtggtgaa |
| RM107 | 9 | agatcgaagcatcgcgcccgag | actgcgtcctctgggttcccgg |
| RM108 | 9 | tctcttgcgcgcacactggcac | cgtgcaccaccaccaccaccac |
| RM219 | 9 | cgtcggatgatgtaaagcct | catatcggcattcgcctg |
| RM242 | 9 | ggccaacgtgtgtatgtctc | tatatgccagcacggatggg |
| RM296 | 9 | cacatggcaccaacctcc | gccaagtcattcactact |
| RM3249 | 9 | gcccttttcttctccactcc | agacactgtcacagcttcagc |
| RM3700 | 9 | aaatgccccatgcacaac | ttgtcagattgtcaccaggg |
| RM3744 | 9 | caggtaagttttcattttca | gagcaggagtaacagttgta |
| RM5657 | 9 | tatgtgcatttgtaaggtga | gctttagattattgagcgag |
| RM6570 | 9 | cgatccgcatctcgaatc | cctccaaggtcctcatcctc |
| RM7175 | 9 | acagtaaacgtggtgcctcc | agaagtagcctcgaggaccc |
| TC124 | 9 | gctcaaagaaaaggtgacgc | ggaacttacaacctgggcaa |
| TC136 | 9 | cgctgcccttgatgaactat | gagcaaactcctccctgatg |
| RM1125 | 10 | ggggccagagttttcttcag | gtacgcgcagaaaatgagag |
| RM1146 | 10 | accccgatgatcgattgtac | ccctattcccgtgtaaatcg |
| RM1236 | 10 | agaaaagttaattccaaagg | caaggaattctagaggagtg |
| RM2125 | 10 | tacctcctagctttacttat | actgatctctatctcattgt |
| RM216 | 10 | gcatggccgatggtaaag | tgtataaaaccacacggcca |
| RM244 | 10 | ccgactgttcgtccttatca | ctgctctcgggtgaacgt |
| RM258 | 10 | tgctgtatgtagctcgcacc | tggccttaaagctgtcgc |
| RM3019 | 10 | atgggtactaacaaagttca | cttcttcgtcatattctttc |
| RM3229 | 10 | cttgcaacttgcaacgtcc | gcatagcaagaggccaagag |
| RM3283 | 10 | cccgttaaaagggaaactcc | cgaactcctagactccaccg |
| RM5373 | 10 | ggagatgctatagcagcagtg | attgctccttaccaccttgc |
| RM6144 | 10 | tggaactcaacgggagtctc | gaagtagtggaatcggcgag |
| RM6824 | 10 | gagagaacctggtggtggag | agtggtagaagatccgagatcg |
| RM7361 | 10 | ggctcaattcgtaggtgcc | ttctccggttaacgtggaag |
| TC17 | 10 | aactaccgatccgtcaccac | cgaagcacctccctgaatag |
| TC41 | 10 | aaaacattttgcacttgccc | ctacaaggtacagcccccaa |
| TC57 | 10 | agaggagggtgtggtgactg | cctaccgtgatgactaaaagcc |
| RM1240 | 11 | ccatgagctagtaactgcagc | ggatcgcaaaatctggcatc |
| RM206 | 11 | cccatgcgtttaactattct | cgttccatcgatccgtatgg |
| RM224 | 11 | atcgatcgatcttcacgagg | tgctataaaaggcattcggg |
| RM254 | 11 | agccccgaataaatccacct | ctggaggagcatttggtagc |
| RM3625 | 11 | cttgcaattcaattgcttac | ggtggcctagtgaaactaaa |
| RM4504 | 11 | taattgatgagcttgatgta | agagagattttatgaaacca |
| RM5349 | 11 | agggcatgcttacatccaac | catttgcttctatgccccag |
| RM5961 | 11 | gtatgctcctcctcacctgc | acatgcgacgtgatgtgaac |
| RM6544 | 11 | accactatgcacccttcgtc | gaatgctctgcttcgtttcc |
| RM6897 | 11 | atatccgatgtgacacgcag | aggataaattgggtggggac |
| TC126 | 11 | caaccagtgcttgagcttga | agtaacagcatccatccatcg |
| TC18 | 11 | tcgtgccgattttaatttcc | agcaggttcaaccaatcagg |
| RM117 | 12 | cgatccattcctgctgctcgcg | cgcccccatgcatgagaagacg |
| RM1227 | 12 | atggtagagacgagagatgg | ggaccactccaacaatttta |
| RM1261 | 12 | gtccatgcccaagacacaac | gttacatcatgggtgacccc |
| RM179 | 12 | ccccattagtccactccaccacc | ccaatcagcctcatgcctcccc |
| RM2197 | 12 | actgagaactttaatcatcg | gaacaactttgaagagaaac |
| RM235 | 12 | agaagctagggctaacgaac | tcacctggtcagcctctttc |
| RM247 | 12 | tagtgccgatcgatgtaacg | catatggttttgacaaagcg |
| RM270 | 12 | ggccgttggttctaaaatc | tgcgcagtatcatcggcgag |
| RM2734 | 12 | gctctactgctctagagcaa | gccacggattaatatatgaa |
| RM3103 | 12 | cagacaacttgtaatgtacg | atgtcatgggagataattaa |
| RM3246 | 12 | gccactcatataagcaaatg | tggttaatggtcagaacctg |
| RM3455 | 12 | tgaatccacactcgcagatc | gccagtccacgattggtc |
| RM3726 | 12 | cacacacatcgctcggtc | gatgtggaggtcgatggc |
| TC19 | 12 | gcggtgaggagagtaactgg | gcttcatctgggaggctaca |
| TC49 | 12 | ccctctttccctcgtcttct | accatctctcccgcctagag |
| KM180 | Unknown | ttccatgcagggatgttgta | gaaggagacttggctcaacg |
| KM182 | Unknown | atgttgcagcagagcatttg | tggggttgttgttgctgata |
| KM186 | Unknown | tgaagaagtggcacaagacg | atggtcttgtagcggtggag |
