# Supplementary material for: Genetic Analysis of Cold Tolerance at the Germination and Booting Stages in Rice by Association Mapping
Source: PLoS One. 2015 Mar 19;10(3):e0120590. doi: 10.1371/journal.pone.0120590 (PMC4366098; doi:10.1371/journal.pone.0120590)
Supplement: S3 Table — (DOC) [file pone.0120590.s004.doc]

**Table S3 LD degrees (r^2^) between markers with different physical distances**

| **Physical distance** | **Whole population** | ***Indica*** | ***Japonica*** |
| --- | --- | --- | --- |
| <50 kb | 0.1443±0.2149 | 0.1388±0.2135 | 0.1810±0.3203 |
| 50-150 kb | 0.1332±0.1829 | 0.0761±0.0796 | 0.0760±0.0368 |
| 150-500 kb | 0.0926±0.1353 | 0.0331±0.0573 | 0.0392±0.0616 |
| 500-1000 kb | 0.0624±0.0765 | 0.0155±0.0148 | 0.0336±0.0505 |
| >1000 kb | 0.0605±0.0818 | 0.0134±0.0165 | 0.0201±0.0261 |
| Genome average | 0.0624±0.0865 | 0.0154±0.0311 | 0.0222±0.0409 |
